# Being as Having, Loving, and Doing: A Theory of Human Well-Being

**DOI:** 10.1177/10888683241263634

**Published:** 2024-07-26

**Authors:** Frank Martela

**Affiliations:** 1 Aalto University, Espoo, Finland

**Keywords:** Erik Allardt, eudaimonic well-being, physical needs, psychological needs, self-determination theory, subjective well-being, well-being

## Abstract

**Academic Abstract:**

Stronger theory on the nature of human well-being is needed, especially as well-being indicators are increasingly utilized in policy contexts. Building on Erik Allardt, who argued that a theory of well-being is, in essence, a theory of human nature, I propose four modes of existence each capturing one dimension central to human well-being: *Having* recognizes humans as biological creatures requiring certain material resources for survival. *Loving* captures human social nature and our dependence on others for well-being. *Doing* highlights the active and agentic nature of human existence. *Being* acknowledges humans as experiencing their existence. Each mode of existence gives rise to a few more specific needs, and a full assessment of human well-being requires both subjective and objective indicators tapping into these needs. The proposed theory integrates psychological well-being research with sociological and philosophical traditions and contributes to debates about how the progress of nations and sustainability should be measured.

**Public Abstract:**

Well-being is something we all value individually, and it is also a key political goal. Accordingly, how we define and measure well-being influences what physicians, managers, policy-makers, politicians, and international organizations aim to improve through their work. Better theories of well-being make better measurement of well-being possible, which makes possible more effective and evidence-based advancement of human well-being. In this spirit, the present article argues that there are four fundamental dimensions to human well-being: *Having* highlights that as biological creatures, we have physical needs, *loving* highlights human social needs, *doing* highlights that we are active and agentic beings with goals and strivings, and *being* highlights that we feel and evaluate our lives. To assess well-being, we need measures tapping into all four of these dimensions. And to assess the *sustainability* of well-being, we need to examine how to provide well-being for all humanity while remaining within planetary boundaries.

## Introduction

Improving well-being is one of the key targets of societies and politics. International organizations from the United Nations (2011) and UNESCO (2024) to the Organization for Economic Co-operation and Development (OECD, 2013) call for the measurement of citizen well-being to be used as targets of politics. Responding to this call, virtually all OECD countries have nowadays some subjective well-being indicators as part of their national statistics (Stone & Krueger, 2018), and over 30 countries including Iceland, France, New Zealand, South Korea, Mexico, and Ecuador have all built their own national well-being frameworks to assess national progress (Exton & Shinwell, 2018; Stone & Krueger, 2018). The importance of measuring well-being more directly has been highlighted as the inadequacy of gross domestic product (GDP), and other economic indicators as measures of development are increasingly recognized because they fail to capture many central areas of human well-being such as social relationships and freedom from oppression while being inflated by factors clearly harmful for well-being such as increased arms production due to wars (Costanza et al., 2014; Hoekstra, 2019; Stiglitz et al., 2009). How to provide well-being for all humanity in an environmentally sustainable way has become the key question of our age (Hickel, 2019; O’Neill et al., 2018; Randers et al., 2019; Raworth, 2017).

Identifying ways to improve human well-being is also a key target and one of the most studied outcomes across many scientific fields such as psychology (reviewed in Clark et al., 2019; Diener, Lucas, & Oishi, 2018), organizational research (Judge et al., 2017; Judge & Klinger, 2008; Wright & Huang, 2012), medicine and public health (Trudel-Fitzgerald et al., 2019; VanderWeele et al., 2020), and increasingly also economics (Clark, 2018; Krueger & Stone, 2014), while in development studies, there is increased recognition that a conception of what well-being is for humans is needed as the common yardstick against which any notion of social progress should be measured (Alkire, 2002; Doyal & Gough, 1984; S. C. White, 2010).

How we measure well-being—what indicators we use to capture it—directly influences what policy-makers, politicians, and international organizations aim to improve through their work, and what scientists across many fields are examining and targeting in their experiments and interventions. Well-being is also a key target for many managers, educators, coaches, and physicians—as well as something most people across the world value and aim for in their own lives (Heine, 2020; Zwolinski, 2019). As Ryan and Deci (2001, p. 142) put it, “how we define well-being influences our practices of government, teaching, therapy, parenting, and preaching, as all such endeavors aim to change humans for the better, and thus require some vision of what ‘the better’ is.”

Measuring human well-being, however, necessitates a theory of well-being. Every indicator of well-being is based on some more or less explicit assumptions about what a good life, wellness, and flourishing are for humans. Yet, despite thousands of empirical studies (Diener et al., 1999; Diener, Oishi, & Tay, 2018), research on well-being has tended to be relatively atheoretical, leading to a large number of competing accounts of well-being (reviewed in Heintzelman, 2018; Marsh et al., 2020; Martela & Sheldon, 2019) and calls to anchor well-being research in more explicit and elaborated theories of what well-being as such is (Alexandrova, 2017; Dodge et al., 2012; Fabian, 2022b). Prinzing (2021, p. 294) puts this in strong terms: “To realize its *raison d’etre*, the field needs to reflect more seriously on what well-being really is. A science of well-being simply must have a clearly articulated, well-considered, publicly discussed vision of the good life.”

In this article, I aim to advance this anchoring of well-being to theory by proposing a theory of well-being, which builds on the classic contribution of Erik Allardt (1976, 1993) and its later refinements (Helne & Hirvilammi, 2015; Hirvilammi & Helne, 2014), integrating it with later research on basic psychological needs and insights from self-determination theory (Martela & Ryan, 2023; Ryan & Deci, 2000, 2017). The starting point for the present theory is the idea that human beings are certain types of creatures—there is a certain human nature that we share as a species—and a theory of *human* well-being must take this human nature into account. Accordingly, the present theory can be seen as a needs-based theory of well-being (Doyal & Gough, 1991; Galtung, 1980; Ryan & Deci, 2017), where needs are understood as factors humans universally need for their survival, functioning, and flourishing.

More particularly, I come to propose that four central modes of existence form the backbone of the human way of existing, and within each, we can identify a few more specific needs (Allardt, 1976). First, humans are biological-material beings, making ***having*** certain physical needs satisfied, such as nutrition and oxygen, necessary for our survival and well-being. Second, humans are inherently social animals, who have social needs, and whose wellness is deeply tied to other people, making ***loving*** another fundamental aspect of human well-being. Third, humans are active and agentic beings, guided in action by inner goals, values, and preferences, making agentic needs associated with ***doing*** a central aspect of human well-being. Fourth, humans experience their existence, having feelings and making evaluations, making ***being*** the most fundamental aspect of human wellness, associated with a general nality Statement should appeeed to value, appreciate, and feel positive about one’s existence as such.

Catchphrases aside, each mode of existence represents a fundamental claim about the type of existence that human life innately is. Within each mode, I will identify a few central needs along with a number of key subjective and objective indicators to measure them. This novel typology thus anchors elements of well-being more deeply in human nature and basic human needs—while making psychological research on subjective well-being more anchored in broader sociological and philosophical discussions on the basic nature of well-being.

Besides identifying the four modes of human existence, I suggest that in policy contexts the sustainability of well-being must also be taken into account to ensure that current well-being is not advanced at the expense of the ability to maintain well-being in the future. This helps to integrate a theory of well-being into key sustainability challenges of our present time (O’Neill et al., 2018; Randers et al., 2019).

Positionality Statement. The author is a Nordic scholar educated in philosophy, psychology, and organizational research in North European universities, with exchange periods spent in Thailand, Australia, and the United States. Thus, notwithstanding conscious attempts to broaden his perspective, he has been mostly exposed to European and North American culture, philosophy, and research.

## The Current Need for Theories of Well-Being

Well-being has many definitions, but on the most general level, it refers to life that is good for the person living that life (Haybron, 2008; Tiberius, 2006). A certain life can be good from the point of view of the nation, from a moral point of view, or from the point of view of contributing to the arts or sciences. Well-being, in contrast, refers to a life that is going well from the point of view of the person in question, a life “which is good for the person whose life it is” (Raz, 2004, p. 269). This means that well-being consists of elements generally seen as good, valuable, and desirable for the given human being. Accordingly, well-being is at the center of theories of what humans want and need in life, and what individuals and societies ought to advance in life (Tiberius, 2006). When we aim to define well-being, we continue an inquiry already central for Aristotle (2012, p. 2), whose *Nicomachean Ethics* is essentially a quest to identify what is “some end of our actions that we wish for an account of itself, the rest being things we wish for on account of this end.”

Dimensions of well-being are, accordingly, by definition aspects of human experience deemed as desirable, valuable, and positive, making any indicator of well-being into a “value-oriented” (Allardt, 1976, p. 227) and “value-laden” (Fabian, 2022b, p. 15) claim of what is good and worth having in life—thus advancing a particular claim about human nature. However, too often indicators of well-being have been developed without making these basic claims about human nature explicit. This “absence of theory-based formulations of well-being” in psychology (Ryff & Keyes, 1995, p. 719) has been long recognized (Dodge et al., 2012; Fabian, 2022b; Forgeard et al., 2011; King et al., 2004). The importance of explicitly linking each proposed indicator of well-being to their underlying theory of human nature is made especially crucial by three recent developments.

First, as noted in the introduction, there is an increased call to take subjective indicators of well-being seriously in policy contexts, as a key way of measuring national progress (Cummins et al., 2003; Diener et al., 2015; Diener & Seligman, 2004, 2018; Dolan & White, 2007; Frijters et al., 2020; Graham et al., 2018). However, the more there have been calls to use subjective indicators of well-being in public policy, the more there have been also critics noting that in making such claims, the researchers on subjective dimensions of well-being “have overstepped its limits” (Fabian, 2022b, p. 2), citing methodological weaknesses (T. N. Bond & Lang, 2019; Fabian et al., 2021) and theoretical immaturity (Alexandrova, 2017) as reasons for why subjective well-being indicators are not yet ready to be used as benchmarks in public policy (Benjamin et al., 2020). To claim that a certain indicator of well-being is truly tapping into what well-being is, we first need to have an explicit theory of well-being (Alexandrova, 2017)—something the present paper aims to provide.

The second reason why more theory on well-being is needed is the proliferation of various competing instruments to measure well-being. Cooke et al. (2016, p. 730) reviewed 42 instruments to measure subjective dimensions of well-being, concluding that “there is considerable disagreement regarding how to properly understand and measure well-being.” Martela and Sheldon (2019), in turn, identified at least 45 different ways of conceptualizing various dimensions of well-being that used measures of at least 63 different constructs. For illustration of this diversity, a few influential measures (e.g., Diener, Wirtz, et al., 2010; Seligman, 2011; Su et al., 2014; Tennant et al., 2007) and their dimensions are listed in Table 1. Table 1 also shows that while all the presently proposed elements have been recognized at least by one theory, none offers a similar conceptualization of the key elements of well-being. Without stronger theory about what well-being itself is and how various proposed elements of well-being relate to each other, we have no way of saying why one measure captures human well-being better than the other, leading to “blurred and overly broad definitions of well-being” (Forgeard et al., 2011, p. 81). This situation where well-being literature currently “lacks a coherent theoretical and methodological framework” (Das et al., 2020, p. 24) is an “untenable situation if the aim is to do comparable and cumulative science” (Martela & Sheldon, 2019, p. 461). The remedy would be stronger theory which can then be used to make more informed selections as regards what elements and measures to use to best tap into human well-being.

**Table 1.** The Subjective Dimensions of Well-Being as Posited by a Number of Influential Theories Arranged According to the Presently Proposed Dimensions.

	Having		Loving			Doing		Being		Other
Well-being theory	Physical needs	Safety	Inclusion	Relatedness	Prosociality	Autonomy	Competence	Affective well-being	Evaluative well-being	
**Subjective well-being** (Diener, 1984)								Positive affect Negative affect	Life satisfaction	
**Psychological well-being** (Ryff, 1989)				Positive relations		Autonomy	Environmental mastery		Purpose in life	Personal growth Self-acceptance
**Basic psychological needs from SDT** (Ryan & Deci, 2000)				Relatedness		Autonomy	Competence			
**Personal Wellbeing Index** Cummins et al., 2003)	Standard of living	Safety Future security		Personal relationships Community connected-ness			Achieve in life			Health
**Psychological functioning in Warwick-Edinburgh mental well-being scale** (Tennant et al., 2007)						Autonomy	Competence	Energy		Clear thinking Self acceptance Personal development
Flourishing Scale (Diener, Wirtz, et al., 2010)			Being respected	Supportive relationships	Contribution to others		Competence	Engagement	Purpose and meaning	Optimism Being a good person
**PERMA theory of well-being** (Seligman, 2011)				Relationships			Accomplishment	Positive emotions Engagement	Meaning	
**Mental Health as Flourishing** (Huppert & So, 2013)				Positive relationships			Competence	Positive emotion Emotional stability Vitality Engagement	Meaning	Optimism Resilience Self-esteem
**Comprehensive Inventory of Thriving** (Su et al., 2014)			Respect Loneliness	Belonging Support Community Trust		Autonomy	Skills Learning Accomplishment Self-efficacy	Positive feelings Negative feelings Engagement	Life satisfaction Meaning and purpose	Optimism Self-worth
**The Well-Being Profile** (Marsh et al., 2020)				Positive relations	Prosocial Empathy	Autonomy	Competence	Positive emotions Vitality Engagement Emotional stability	Meaning	Self-esteem Self-acceptance Clear thinking Optimism Resilience

The third reason why stronger theories of well-being are needed is the increased recognition of the narrow Western origins of most mainstream psychology (Apicella et al., 2020; Henrich et al., 2010a, 2010b). In a globalized world, we need a global psychology (Berry, 2013)—especially if we want to be able to compare the well-being of people across the world. While cultural differences exist and influence well-being, all humans share the same human nature (Mathews & Izquierdo, 2010; Zwolinski, 2019). Accordingly, anchoring accounts of well-being to this shared human nature would be a way to overcome the Western bias in psychology. Identifying the shared human needs beyond cultural differences is not an easy task, requiring extensive cross-cultural research programs, but engaging in this task is necessary, if we want to recognize the universal factors of well-being that unite humans across borders (Berry, 2013; Jensen, 2012).

## Four Modes of Existence: Having, Loving, Doing, and Being

Building a theory of well-being starts with building an understanding of human nature. When the social indicators movement emerged on the international scene in the 1960s and 1970s, the focus was typically on objective indicators examining the resources available to the individual, such as education, employment, family relationships, financial resources, and political resources (e.g., Johansson, 1973; see Land & Michalos, 2018). Against this backdrop, sociologist Erik Allardt (1925–2020) argued, partly inspired by Maslow (1943), that human beings are more than material creatures, and thus a mere focus on material living conditions (having) is not enough. In particular, he emphasized that humans have also social needs (loving) and needs related to self-actualization and personal growth (what I here call doing), which must be taken into account for a more comprehensive understanding of well-being (Allardt & Uusitalo, 1972). Furthermore, building on the research on subjective assessment of well-being that has proliferated since Allardt’s time (Diener, Oishi, & Tay, 2018; Krueger & Stone, 2014), a key dimension of well-being is how humans in general experience and evaluate their own lives (being).^1^ There is more to human existence than mere survival, we are social and active beings who want to find value in our own lives, and all these dimensions should be examined when assessing human well-being and how well a person’s life is going. Each need category aims to capture some “central necessary conditions of human development and existence” (Allardt, 1993, p. 89).

Even more fundamentally, I argue that we should see having, loving, doing, and being as separate *modes of existence* or *modes of being*, each emphasizing one way humans exist in the world. With a mode of existence, I thus mean a basic way of *being in* and *relating to* the world. Modes of existence does not answer the question of *whether* humans exist but rather the question of *how* humans exist and what exactly is our “existential situation” (Dewey, 1938, p. 105). An inquiry into the basic modes of existence is a phenomenological investigation of the key essences of human experience and human way of being (cf. Heidegger, 1962), what John Dewey called a “cognizance of the generic traits of existence” (Dewey, 1925, p. 50), where we researchers are “human beings striving to comprehend a world of human experience by the resources of human minds” (Schiller, 1907, p. 12), aiming to discern the different ways of existing characteristic of this irreducibly human way of being in the world (Martela, 2015).

The four recognized modes of existence identify human ways of being in the world. But taken as such, they remain on a rather abstract level. To make them more concrete and measurable, we can discern more specific human needs within each mode. Having, loving, doing, and being should thus not be seen as basic needs but represent a “classification of basic needs” based on their mode of satisfaction (Allardt, 1976, p. 231), with each class potentially containing several more specific needs.

Human needs are here defined as factors humans universally need for their survival, functioning, and flourishing. Needs reflect “our adaptive human design” (Ryan & Deci, 2017, p. 88), and a degree of need satisfaction is necessary for the subject “to function as a human being” (Galtung, 1980, p. 60), with need frustration associated with serious harm (Allardt, 1976). Needs are typically contrasted with wants and wishes, which are subjectively felt and articulated and vary between individuals (Allardt, 1976; Galtung, 1980). Unlike wants, needs are universal in the sense of being the same for all humans. Furthermore, needs do not have to be subjectively articulated—their satisfaction impacts our well-being whether or not we are aware of them (Ryan & Deci, 2017). In fact, a person’s wishes, goals, and wants can be more or less in line with their needs—and this alignment with needs typically has well-being consequences (Bradshaw et al., 2023; Niemiec et al., 2009). Thus, the most important criteria for a basic human need are that (a) the satisfaction of the need should be directly connected to positive affective consequences and long-term functional benefits. (b) The frustration of the need should be directly connected to negative affective consequences and long-term functional harm. (c) The need should be universally operational across cultural contexts and developmental periods, regardless of individual preference (Martela & Ryan, 2023; Ryan & Deci, 2017; Vansteenkiste et al., 2020).

Next, I will make the case for and define each of the four modes of existence, aiming also to identify the more specific needs and themes within each of the four modes. I will also propose both subjective and objective indicators for each (summarized in Table 2). Besides subjective need satisfaction, it is important to identify and measure objective *need satisfiers* (Max-Neef et al., 1992), which refer to the various external resources and conditions that give rise to need satisfaction (Allardt, 1976). Furthermore, different objective need satisfiers can be “at different distances” from well-being as such (Allardt & Uusitalo, 1972, p. 19), with direct satisfiers focusing on factors available to the individual in question, and indirect satisfiers to institutional and structural factors that support individual-level need satisfaction. Given the conceptual nature of the present inquiry, the focus will be on identifying and justifying the key constructs to be measured within each mode of existence, rather than discussing the more specific scales and indicators to be used to measure them. Accordingly, especially the objective indicators will be listed quite briefly.

**Table 2.** A Preliminary Proposal of the Key Objective and Subjective Indicators for Having, Loving, Doing, and Being.

Mode of existence	Basic needs and key dimensions	Subjective indicators	Direct need satisfiers	Indirect, institutional need satisfiers
**Having**	**Physical needs**	Perceived access to water	Access to clean drinking water	Crime and homicide rates
	**Oxygen**	Perceived access to food	Access to food (malnutrition)	Social security system
	**Hydration**	Satisfaction with sleep	Access to sanitation	
	**Nutrition**	Satisfaction with living conditions	Opportunity to sleep	
	**Sleep**	Sense of security as regards employment	Access to housing	
	**Shelter & protection**	Satisfaction with the adequacy of income	Victim of crimes, hazards & violence	
	**Safety and security**	Sense of safety and security	Employment (unemployment rate, decent working conditions)	
			Income (poverty rates)	
			Availability of social security benefits	
**Loving**	**Inclusion**	Sense of inclusion and lack of discrimination	Family status	Indicators of discrimination
	**Relatedness**		Number of close friends	Indicators of segregation
	**Prosociality**	Sense of relatedness and mutually caring relationships	Frequency of prosocial behavior	
		Sense of prosociality		
**Doing**	**Autonomy**	Sense of autonomy	Leisure time and work-life balance	Freedom of choice and expression
	**Competence**	Sense of competence	Educational level	Political rights
				Possibilities for civic engagement
				Gender equality
				Other types of social equality
				Access to education (literacy rate)
**Being**	**Affective well-being**	Positive affects	General physical health	Access to health care
	**Evaluative well-being**	Negative affects	General mental health	Life expectancy
		Life satisfaction	Physical functioning	
		Meaning in life		

### Having: Humans as Biological-Material Beings

Humans are biological and material creatures whose continuous existence is dependent on getting certain physical resources from the environment. We need oxygen to breathe, we need water to drink, we need nutrition, we need sleep, and we need shelter from too much heat, cold, direct sunshine, predators, and other threats to our physical survival. These physical needs (Maslow, 1943) should form a backbone for any examination of human well-being, as a serious lack of any of these could be fatal for the individual. *Having* as a need category captures such needs, as it is about “needs related to material and impersonal resources” (Allardt, 1976, p. 231)—the various material factors we need to survive and not suffer.

Physical needs typically adhere to a homeostasis model, where they become “salient primarily when the individual does not have it” (Ryan & Deci, 2017, p. 251), activating motivation and behavior to bring about a satiated state of balance (Maslow, 1954). They thus have the simple evolutionary function of motivating the organism to seek out all the material resources necessary for survival (Kenrick et al., 2010). Serious deficits in any of these crucial material resources heavily undermine the quality of life and well-being (Biswas-Diener & Diener, 2006), causing suffering, no matter how much excess there is as regards other needs, emphasizing the necessity of each of our basic material needs (Maslow, 1954). Research utilizing a sample of 123 countries showed that deficits in such basic physical needs is strongly correlated with lower life satisfaction and more negative emotions across the world (Tay & Diener, 2011; see also Diener, Ng, et al., 2010).

The relevant objective indicators to measure physical need satisfaction include access to key need satisfiers, such as *access to clean drinking water, access to sanitation, access to food* (level of malnutrition), *opportunity to sleep, access to housing* (living standards, access to adequately protective housing, homelessness), and whether the person has been the *victim of various hazards, crimes or violence*—from other individuals or state institutions. Given that income received from work is a key means to satisfy most physical needs in modern societies, indicators of *employment* (employment rate, unemployment rate, access to work with decent conditions) and *income* are relevant, although here poverty rates are more relevant than average income levels, as excessive incomes tend to have diminishing returns on need satisfaction and well-being (Howell & Howell, 2008; Jebb et al., 2018). These threats that lack of income and unemployment have toward physical need satisfaction are, however, mediated by the social security system provided by the government: A functional safety net can mitigate such problems. Relevant to the satisfaction of physical needs is thus also the availability of *social security benefits* and aid for the individuals. In regards to more indirect, institutional-level need satisfiers, *crime and homicide rates* and *the availability of social safety net* are key indirect indicators of whether the safety and physical needs of the person are threatened.

These objective indicators can be complemented by asking respondents about perceived threats to the satisfaction of these physical needs, as some physical need being threatened might not be revealed by the objective indicator capturing its current presence. Given the role of money in satisfying physical needs, it is also important to ask the subjects about *the adequacy of their income* and *the security of their employment*.

Beyond these more specific concerns, one should also examine a more general *sense of safety and security* in life, meaning a need to ensure that we are in a relatively safe and secure place, protected from any immediate or impending threat. Maslow (1943) proposed that along with purely physical needs, humans have a need for safety, and Allardt saw that both are related to *having* (Allardt & Uusitalo, 1972), as safety is also a *deficit need*, becoming mainly activated when something threatens it, and having a strong motivational pull in the face of various dangers, which leads it to override other concerns. Later research inspired by Maslow has confirmed that a lack of safety is associated with increased ill-being and people feeling threatened, which often has detrimental consequences (Chen, Van Assche, et al., 2015; Rasskazova et al., 2016). Cross-cultural research, in turn, has demonstrated the importance of subjective sense of safety for well-being, even when controlling for other factors such as typically recognized psychological needs (Tay & Diener, 2011), with Gallup having gathered data on subjective sense of safety in 140 countries (Gallup Inc., 2023b). A subjective sense of safety can function as an overall subjective assessment of how well the individual feels when they are protected against various threats to survival and potential deficits in their physical needs.

### Loving: Humans as Social Beings

Humans are social animals. We are born into a network of relationships, we develop through them, we become aware of and define ourselves through them, we seek out others and constantly interact with them, and we are dependent on others for our survival and well-being. Each human being needs reciprocal relations with people one “cares for” and by whom one is “cared for” (Allardt, 1973b, p. 65). *Loving* as a need category captures the human need to feel they have high-quality relationships with, belong to, and are accepted by relevant social groups (Allardt, 1976; Baumeister & Leary, 1995; Deci & Ryan, 2000), being about “needs related to love, companionship, and solidarity” (Allardt, 1976, p. 231). This need to belong and have caring social relationships is recognized by virtually all need theories (Alderfer, 1969; Bakan, 1966; Baumeister & Leary, 1995; Maslow, 1943; McClelland, 1985; Ryan & Deci, 2017), and also anthropological accounts tend to emphasize the importance of relationships for humans (Boehm, 2001; Brown, 1991). I see that this sociality is so deeply entrenched in the human way of being that we can see sociality as a mode of existence for humans; our way of existing is existing in and through social relationships.

Given the importance of sociality for humans, it is likely that our social needs do not reduce to one—or at least that within this one broad need, there might be identifiable sub-needs. Thus, there might be a few qualitatively different human social needs. First, at a minimal level, it is important to be accepted by one’s relevant social groups. Being discriminated against, feeling like one does not fit in, or being outright rejected and ostracized have been shown to be highly painful experiences (e.g., Eisenberger et al., 2003; Legate et al., 2013), associated with depression and mortality, among other negative effects (Hawkley & Cacioppo, 2010; Williams, 2007). Research has shown that experiencing discrimination—not receiving equality of treatment and being subject to devaluation and prejudices—based on one’s ethnicity (Benner et al., 2018; Fryberg et al., 2008), gender (Schmitt et al., 2002), sexual orientation (Jackson et al., 2019), or disability (Hackett et al., 2020) is associated with ill-being, such as distress and depression, particularly when one is part of a disadvantaged group (Schmitt et al., 2014) or when one experiences multiple forms of discrimination (Vargas et al., 2020). Accordingly, the avoidance of social exclusion is a strong human motive (Martela et al., 2019; Williams, 2009), with research around the Asch paradigm having shown how humans are willing to go to great lengths to ensure that they fit into the group (e.g., Asch, 1956), with replications in countries on all continents showing that conformity is especially prevalent in collectivist countries (R. Bond & Smith, 1996). Given that human survival in both prehistorical and later eras has been intimately tied to group membership, social exclusion has tended to be a life-threatening risk (Boehm, 2001; Wesselmann et al., 2012). This evolutionary pressure has made humans highly sensitive to the threat of being ostracized (Kerr & Levine, 2008; Spoor & Williams, 2007), giving rise to the defensive need to ensure that one is accepted by others. This need for acceptance arguably functions mostly as a deficit need—its lack causing ill-being. Accordingly, a comprehensive well-being assessment should ask subjects about their *sense of inclusion* and lack of discrimination at the local as well as national levels.

However, beyond being merely accepted by others, humans need caring mutual relationships. Accordingly, a need for relatedness as a “desire to feel connected to others” and to have mutually caring relations has been postulated as a universal psychological need for humans (Deci & Ryan, 2000, p. 231), with a broad body of research, including cross-cultural comparisons (e.g., Chen, Vansteenkiste, et al., 2015; Church et al., 2013), demonstrating its importance for human well-being (reviewed in Ryan & Deci, 2017; Vansteenkiste et al., 2020). This need is satisfied by the presence of relationships characterized by care, intimacy, mutuality, and a sense of connection—what could be characterized as high-quality connections (Dutton & Heaphy, 2003). Assessing *relatedness* as the sense of mutually caring relationships is included in the European Social Survey Well-being Module (Huppert et al., 2009), but otherwise, it has rarely been part of national well-being assessments, although OECD recommends measuring it more in the future (Mahoney, 2023).

Third, in addition to feeling a sense of connection and being cared for by others, humans seem to have a range of prosocial motivations, making them care about the well-being of others and want to have a positive impact in the lives of others (Batson, 1990; Lishner et al., 2011; Martela & Ryan, 2016a; Singer & Klimecki, 2014). Doing good to others thus seems to be a salient motive for humans, with experimental studies demonstrating that its satisfaction consistently improves our sense of well-being (Curry et al., 2018; Hui et al., 2020; Martela & Ryan, 2016b). Based on cross-sectional evidence from 136 countries and experimental studies from countries on four continents, Aknin et al. (2013, 2015) thus proposed that the well-being benefits of a prosocial behavior are a psychological universal. To assess *subjective prosociality*, respondents can report how much they care about others and have prosocial motivations toward them and how strong is the sense of prosocial impact they experience.

Key objective indicators for *loving* include *family status* and *the number of close friends*, as family and friends are key satisfiers of social needs for most people around the world. One can also track various forms of *prosocial behavior* such as volunteering, charity donations, or provision of informal care. On a societal level, segregation understood as “the degree to which different groups occupy different social environments” (Alonso-Villar & Del Río, 2017, p. 269) is one key manifestation of structural discrimination that significantly affects the life outcomes of various social groups (Massey et al., 1987). This makes *discrimination* and *segregation* important indirect indicators of how well various groups of people are accepted and appreciated in society.

Accordingly, although an exact theory of the key needs related to loving and human sociality would require its own work, I see that in building indicators of human social needs, at least inclusion, relatedness, and prosociality should be recognized as three central facets of such a socially oriented need of humans.

### Doing: Humans as Active Beings

Human relation to the world is active and agentic—we are not mere observers nor do we just passively react to the environmental stimuli. Instead, we actively engage with our lives and have desires and goals, understood as internal representations of preferred future states, that give direction to our activities (Deci & Ryan, 2000). Humans are “not simply onlookers of their behavior” but intentionally and proactively influence their functioning and life circumstances (Bandura, 2006, p. 164). Agency is a universal human quality (Markus & Kitayama, 2003),^2^ denoting a capacity to act purposively and actualize some possibilities instead of others, thus implying a capacity to make choices and a capacity to take action (Frankfurt, 1978). This involves two claims about human existence: First, humans have future-oriented desires, goals, and aspirations that motivate their actions. Second, humans can make choices that orient them toward the fulfillment of their aspirations. *Doing* as a mode of existence captures the active nature of humans as beings capable of making choices and steering their lives based on goals, values, and desires that they find valuable.^3^

A key dimension of human well-being is thus how well we are able to satisfy our agentic needs in our lives: Building on self-determination theory (Ryan & Deci, 2017), I propose two such needs: competence and autonomy, which represent the two key sides of human agency: actions needing to be effective and self-endorsed. First, the *quality of how we act* in the sense of being able to get where we want to get matters to us. This gives rise to the need for *competence*, understood as a sense of effectance, efficacy, and mastery (Ryan & Deci, 2017). Given that humans have goals, aspirations, and responsibilities, they want to feel that they are able to make progress toward their goals, master their activities, and be successful in fulfilling their roles and responsibilities (Bakan, 1966), instead of experiencing failures, ineffectance, and helplessness, that diminish one’s sense of agency. Successfully fulfilling one’s role in society is important for this sense of mastery, with taking up work-related roles leading to personality maturity across the world in a study from 62 nations (Bleidorn et al., 2013). Such a sense of competence and efficacy has been recognized as a key part of human agency (Bakan, 1966) and a fundamental source of motivation (Bandura, 1977; R. W. White, 1959), giving rise to more intrinsic motivation toward an activity (Deci & Ryan, 2000), and being associated with a higher sense of well-being (Ryan & Deci, 2017), having relatively consistent associations with life satisfaction and positive feelings in a cross-cultural study of 123 countries (Tay & Diener, 2011).

Furthermore, humans want to ensure that their activities contribute toward aspirations and values they find valuable and worth pursuing. This gives rise to the second agency-related need, *autonomy*, which is about a sense of volition, self-endorsement, and an internal locus of causality (Deci & Ryan, 2000), thus being closely related to self-integration, self-expression (Alkire, 2002), and self-actualization, in opposition to alienation (Allardt, 1973a). This means a need to feel that one’s actions are self-endorsed, and in line with goals, interests, values, roles, and aspirations one finds valuable, in contrast to coercive acting where what one does is externally imposed and controlled. A sense of autonomy has been found to be energizing, giving rise to well-being and more intrinsic forms of motivation (Ryan & Deci, 2017; Stanley et al., 2021; Van den Broeck et al., 2016), also in cross-cultural studies (reviewed in Chirkov, 2011). Both competence and autonomy are relatively rarely included in national accounts of well-being, although the Well-being Module of the European Social Survey assesses them (Huppert et al., 2009), and the OECD recommends that they should be included in future national accounts of well-being (Mahoney, 2023).

It is important to emphasize that autonomy is not about independence, individualism, or separation from others (Chirkov, 2014; Ryan, 1993), but “people can act autonomously in accord with the communal good” (Markus & Kitayama, 2003, p. 17) and as part of a group, as long as one has a sense of self-endorsement toward the group activities and one’s group membership (Chirkov et al., 2003). Autonomy is essentially about self-endorsement of behavior and its alignment with what one wants and values—be the source of one’s wishes and values personal choice or alignment with in-group values and responsibilities. While making individual choices tends to be especially highly valued in individualistic cultures (Savani et al., 2008), in collectivistic cultures, people tend to be more willing to endorse choices made for them by others, provided that the choice was made by a trusted in-group member (Iyengar & Lepper, 1999), finding value in being able to fulfill one’s role and responsibilities in the group (Miller et al., 2011; Oishi & Diener, 2001). While personhood and agency can be experienced as more separate or—like in African psychology (Adjei, 2019)—more entangled and interconnected with the community, in both cases, one is looking for ways to operate that are in line with what one values and wants to strive toward (Miller et al., 2011; Oishi & Diener, 2001). Accordingly, World Values Survey data from 60+ countries across the world have shown that autonomy is associated with well-being (Bavetta et al., 2017; Verme, 2009), cross-cultural research studies have found that autonomy predicts student achievement (Nalipay et al., 2020), and comparisons of the well-being effects have shown that autonomy predicts well-being roughly equally across the world (Chirkov et al., 2003, 2005; Yu et al., 2018). Endorsement of one’s action and situation by the self is thus at the core of autonomy—be that self independent or interdependent.

The relevant objective indicators for *doing* include *the amount of leisure time and work-life balance* that tap into people’s ability to live the kind of life they want to live and *level of education*, as acquiring education can seriously increase a person’s ability to exercise environmental mastery. For example, illiteracy seriously constrains an individual’s abilities in modern societies. In regards to the relevant indirect society-level indicators, they relate to how well various negative liberties (“freedoms from”) are protected in people’s lives (Berlin, 1969), allowing them to exercise their agency in a self-endorsed way. Accordingly, *freedom of choice and expression, political rights*, and *civic engagement* provide important indicators of the freedom citizens have in society to be themselves and express themselves. Furthermore, inequality, where certain people have fewer rights and freedoms than others, can be a serious obstacle for the oppressed group’s ability to live the life they want to live. Accordingly, *gender equality* and other types of *social equality* are important indicators to ensure that the whole population is able to enjoy freedom to determine how they live their lives. Furthermore, indicators for the *educational level* within a country (illiteracy, educational degrees, objective tests) are important for the more positive liberty and sense of mastery and agency of citizens in that country, although the contribution of education to well-being is nuanced and can vary depending on the economic situation of the country (Desjardins, 2008; Petrakis & Stamatakis, 2002).

### Being: Humans as Experiencing Beings

Humans experience their lives. Being alive and conscious means having experiences and living through a constantly unfolding “stream of experiencing” (Martela, 2015, p. 197), or what William James (1907, p. 107) calls “the flux of our sensations.” In a way, being is the most fundamental mode of existence (cf. Heidegger, 1962). Depending on the situation, we can be more or less social, more or less agentic, more or less oriented toward any material concerns, but as long as we are conscious, we can never stop experiencing. While other modes of existence are associated with more specific physical or psychological needs, being is associated with the more general feelings and evaluations we can have about life and how positive they are. Thus, being is the most basic and content-independent mode of existence, fundamentally present in our lives to the degree that living (as a conscious creature) can be equated with experiencing. As such, it is also the most fundamental dimension of human well-being.

One way of conceptualizing this experiencing is through the dual-process theories of cognition (Epstein, 2010; Evans, 2008; Evans & Stanovich, 2013; Kahneman, 2003), which distinguish between two qualitatively distinct forms of information processing and experiencing. First, we share with many animals the ability for fast, associative information processing operating largely outside of cognitive awareness, which phenomenologically gives rise to various hunches, feelings, emotions, and other non-verbal emotionally charged experiences. However, what distinguishes humans from other animals is our exceptionally developed capability for reflective, consciously monitored, and mostly language-mediated information processing, which phenomenologically is associated with conscious thinking and evaluations. Although in many ways intertwined and in constant interaction (Epstein, 2010; Evans & Stanovich, 2013), our way of being thus involves both an engaged self with feelings and a reflective self, capable of evaluations.^4^

Acknowledging human experiencing as involving both feelings within life and evaluations about life gives rise to two types of well-being: affective well-being and evaluative well-being (Kahneman & Riis, 2005; National Research Council, 2013).^5^ First, human general feelings toward life are most often captured through examining how much people experience various emotions, feelings, and affects. *Affective well-being* is a subjective indicator aiming to capture “peoples’ everyday emotions, their joys, miseries, and pains” (Stone et al., 2018, p. 360). Most typically, a distinction is made between positive and negative affects, as research has shown them to be relatively independent, and thus both sides are needed to more fully capture the human general feelings in life (Bradburn, 1969; Diener et al., 1999). Naturally, more granular distinctions can also be made among affects, such as between high arousal and low arousal affects (e.g., Russell, 1980; Yik et al., 1999)^6^, but in general, happiness is “a universal emotion” that people everywhere value (Heine, 2020, p. 404), with 52% of OECD countries measuring affect as part of their national well-being approach (Mahoney, 2023) and Gallup Global Emotions Report providing assessment of emotions in 142 countries (Gallup Inc., 2023a).

However, human cognitive capacity makes it possible to not only feel life but to reflect upon it and evaluate it, this being a crucial part of our overall experience and way of relating with life. *Evaluative well-being* aims to capture this dimension, being about a reflective assessment of a person’s life as a whole (Graham et al., 2018; OECD, 2013). It has been shown to be empirically separate from the more affective dimensions of well-being (Andrews & Withey, 1976; Diener et al., 1999) and is most often captured with general life evaluation and life satisfaction measures that are now standard parts of many national and international surveys, generally seen as quite universally applicable (Veenhoven, 2008). For example, the World Values Survey has assessed life satisfaction in around 90 countries and the Gallup World Poll in 146 countries across the world, and it is also included in almost all national initiatives to measure subjective well-being (Mahoney, 2023). Humans also want to find meaning and purpose in their lives (Frankl, 1963; King & Hicks, 2021; Martela & Steger, 2016), and thus, a sense of meaning and purpose in life is another general type of life evaluation (Martela & Ryan, 2023) that is included in many national and international surveys on well-being (Mahoney, 2023). One of the advantages of such broad life evaluation measures is that they give “each individual the right to decide” how good they find their own life to be, based on their own standards (Diener, 2000, p. 34), being “content-free” in the sense of referring to the person’s life as a whole rather than a particular aspect of it (Sheldon, 2018).

Parallel to subjective indicators for *being* focusing on the most general aspects of our subjective experience, the objective indicators should also focus on the most general aspects of our objective existence as such. In this sense, *life expectancy and mortality* is a key aspect of being, as death is the moment when our existence ends. Furthermore, indicators for *general physical health, general mental health*, and *physical functioning* are important, as each seriously affects the quality of our being and what we are able to do and be in life. Finally, on the institutional level, *access to health care* significantly impacts the mortality and physical health of citizens and whether they get the necessary help when these are threatened, thus offering a critical indirect need satisfier for being.

### Having, Loving, and Doing as Contributing to Being

Of the modes of existence, *having* is mostly concerned with the sphere of physical needs—with the factors necessary for physical survival. *Doing* and *loving* focus on human psychological needs, covering “functioning well both personally and socially” (Marsh et al., 2020, p. 295), *doing* being about human agentic needs, and *loving* being about human social needs. *Being*, in contrast, is directly about perceived well-being—how well the person is feeling and how they generally evaluate their lives, and as such is not directly associated with any more specific needs.

However, human perceived well-being is to a large degree determined by how much one’s physical and psychological needs are satisfied. Metaphorically, needs identify what humans need to do well in life, while perceived well-being functions as a kind of thermometer that expresses how much humans have gotten what they need to do well. Thus, the effects of various need satisfiers—material resources, social relations, and freedoms and capabilities—on perceived well-being should be to a large degree mediated by need satisfaction (Ryan & Deci, 2017). Perceived well-being, typically measured with life satisfaction and positive and negative affects (Diener et al., 1999), thus functions as a key outcome of need satisfaction, and this contribution to perceived well-being is a key empirical criterion for what counts as a need (Martela & Ryan, 2023; Martela & Sheldon, 2019). Supporting this direction of influence, experimental (e.g., Sheldon et al., 2010; Sheldon & Filak, 2008) and longitudinal studies (e.g., Sheldon & Elliot, 1999; Tian et al., 2014; Unanue et al., 2024) have shown that the satisfaction of the psychological needs for autonomy, competence, and relatedness predicts subsequent well-being, and longitudinal three-wave mediation studies have shown that these three needs mediate the influence of various environmental conditions like materialism (Wang et al., 2017) and supportive teaching style (Jang et al., 2016) on well-being (for reviews, see Ryan & Deci, 2017; Vansteenkiste et al., 2020). This applies also as regards objective indicators of being: Deficits in physical needs directly endangers our health and even survival, and similarly lack of relatedness (Holt-Lunstad et al., 2015) and lack of autonomy (Dalgard & Håheim, 1998; Kasser & Ryan, 1999) have been shown to contribute to increased mortality.

**Figure 1.**
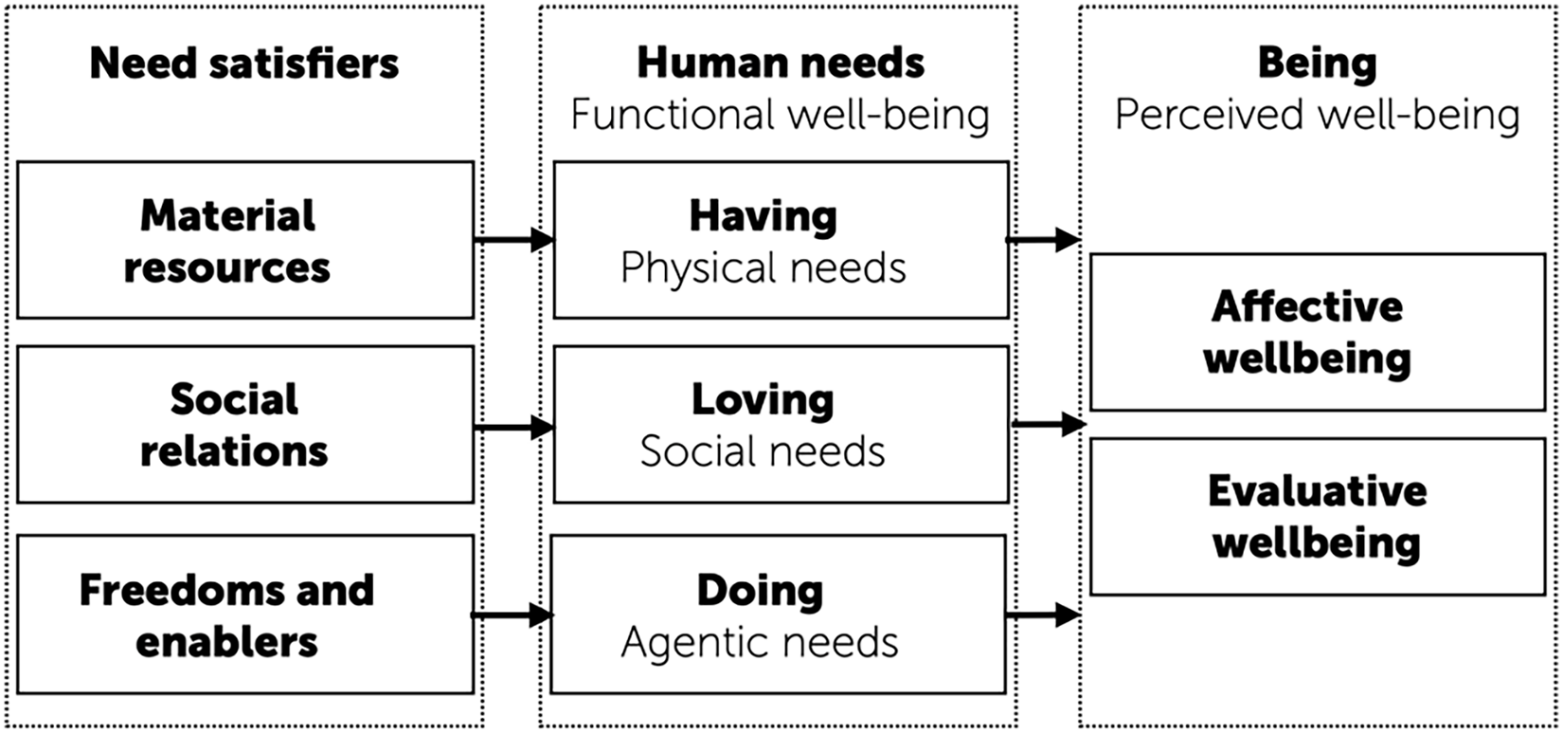
A Schematic Overview of the Relations Between Need Satisfiers, Human Needs, and Perceived Well-Being.

This schematic overview of the relations between need satisfiers, human needs, and perceived well-being is depicted in Figure 1. Note that the figure captures only the main lines of influence, as the reality is more complicated with various reciprocal and dynamic interactions between various elements. The figure highlights how need satisfaction is important for well-being in two ways: First, the satisfaction of human needs leads to perceived well-being, and thus needs operate as key explanations for why a person’s perceived well-being is high or low. Second, as key elements of human physical and psychological functioning, the satisfaction of needs is good for humans as such, beyond their effect on perceived well-being, as they are key factors of human functional well-being. While perceived well-being captures well-*being*, human needs can be said to capture well-*doing*, in the sense of how well the person is physically and psychologically functioning (Huppert et al., 2009; Martela & Sheldon, 2019).

## Why Both Subjective and Objective Indicators Are Needed for Comprehensive Assessment of Well-Being

While psychology has typically focused on subjective indicators of well-being (e.g., Diener et al., 2015), and economics and development studies have traditionally focused on objective indicators of well-being (e.g., Anand & Sen, 2000), I have here proposed both subjective and objective indicators for each mode of being, as there are good rationales for the inclusion of both.

As for the necessity of subjective indicators, well-being is about how well the person in question is doing. Accordingly, we should listen to that particular person when evaluating whether their well-being is high or low, making it crucial to measure well-being with subjective indicators. Against the focus on material conditions popular during his time, Allardt (1993, p. 92) warned about the “dogmatism of experts,” where policy-makers or researchers ignore people’s own opinions, instead determining for them what external factors they need for their own lives to be good. A person high on all objective indicators of well-being can nevertheless be deeply depressed for reasons not captured by these objective indicators. For example, besides absolute poverty, relative poverty (Fritzell et al., 2015) and subjective poverty (Xu et al., 2023) have been found to predict increased mortality. Ignoring the subjective point of view can lead to a significant misclassification of the person’s well-being, as objective indicators are never able to fully capture how well a subject is actually doing. People are typically the best informants in matters related to their own well-being.

However, we should acknowledge that subjective indicators have their own shortcomings and biases. People’s life evaluations are typically bounded by their current situation and the options and alternatives they see available as points of comparison (Allardt, 1976). There is evidence that people engage in scale norming, where their interpretation of the points on a scale changes over time (Fabian, 2022a). For example, a natural experiment involving migrants moving from Tonga to New Zealand showed a lack of improvement in their subjective well-being even when the migrants themselves saw it as having improved (Stillman et al., 2015). These and other methodological challenges (see Benjamin et al., 2020; T. N. Bond & Lang, 2019; Tay et al., 2021), such as the discrepancy between perceived improvement in well-being and actual improvement in well-being measured over time (Prati & Senik, 2022), have made many experts wary of exclusive focus on subjective indicators of well-being as they may be “masking a real improvement in subjectively-assessed welfare” (Fabian, 2022a, p. 1510).

Accordingly, besides subjective indicators, a comprehensive account of well-being should include also some objective indicators. Taken alone, both subjective and objective indicators have their shortcomings and blind spots that the simultaneous measurement of both will help to identify and, to some degree, correct for. Furthermore, the objective indicators can provide invaluable information about the presence of various need satisfiers (Max-Neef et al., 1992) and their impact on well-being.

## From Individual Well-Being to Tracking the Sustainability of Well-Being

The focus of the present article has been on elaborating what well-being is for humans, entailing a focus on the individual. However, it is clear that human well-being does not take place in isolation, but is complexly entwined with social and environmental factors, calling for approaches to well-being acknowledging its complex relational and ecological links and implications (Helne & Hirvilammi, 2017). Especially when thinking about well-being as a societal goal, it is crucial to monitor not only current levels of well-being on an individual level but also key factors giving rise to that well-being that ensure its sustainability. This means that the measurement of well-being as such must be complemented by the measurement of its ecological, economic, and social sustainability (cf. World Comission on Environment and Development [WCED], 1987).

First, we need to monitor ecological indicators and the “quality of the biological and physical environment” (Allardt, 1993, p. 90) to ensure that current well-being is produced in ways that are sustainable as regards environmental systems, biodiversity, and the earth’s limited natural resources. Given that human activities have become the main driver of global environmental change (Rockström et al., 2009), transcending several planetary boundaries and causing major disruptions to earth’s biosphere (Lade et al., 2020; Steffen et al., 2015), monitoring ecological sustainability is an elementary part of any analysis of sustainable human well-being and vital for the survival of both humans, animals, and ecological systems.

Second, individual lives and well-being are deeply embedded in the social and societal context, making social sustainability another key area to be monitored (Littig & Griessler, 2005; Vallance et al., 2011). This involves, first, a communal dimension that tracks the quality of how people relate to each other, including factors such as a sense of trust and respect between people, social capital, and equality between various groups (Dempsey et al., 2011), and second, an institutional dimension-tracking factors related to how the institutions of the society function in protecting and serving the citizens. International comparisons have demonstrated how social capital and trust between people (Delhey & Dragolov, 2016; Helliwell, Huang, & Wang, 2018), gender equality (Audette et al., 2019), and the quality of governance and democratic institutions (Bjørnskov & Tsai, 2015; Helliwell & Huang, 2008; Helliwell, Huang, Grover, & Wang, 2018; Ott, 2011) are substantially associated with the subjective well-being of the citizens. Thus, communal and institutional qualities provide key measures to track the social factors contributing to both the present well-being and the sustainability of that well-being.

Third, given the role of economics as a principal way of organizing the stocks and flows of material and financial resources and labor, it is important to monitor economic sustainability with indicators such as debt, inflation, employment rates, and Gross Domestic Production, to ensure that current ways of producing and ensuring well-being are economically sustainable. However, while human well-being is clearly something worth advancing as such, and the case can also be made for environmental factors such as biodiversity and some dimensions of social sustainability such as fairness, justice, and equality to be basic human values worth defending as such (e.g., Rawls, 1971; Sen, 2009), the economic system has value only to the degree that it advances human well-being. Economics is a system of transforming environmental resources and human labor into human well-being. Thus, it is important to track its functioning and sustainability, but only for instrumental reasons.

**Figure 2.**
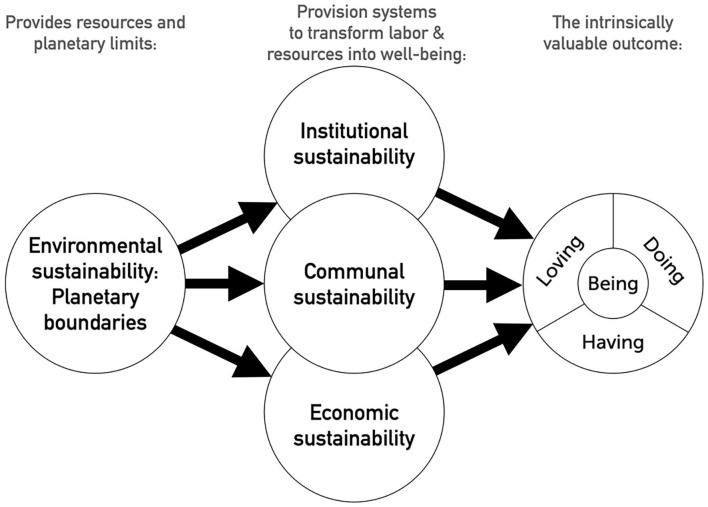
A Rough Characterization of the Relations Between Well-Being and Types of Sustainability.

Accordingly, in addition to monitoring human well-being as such, it is advisable to monitor the sustainability of that well-being through tracking ecological, economic, and social (communal and institutional) sustainability. However, they should not be seen as three separate goals that need to be balanced. Following the Ends-Means Spectrum (O’Neill et al., 2018) and “safe and just space” (Raworth, 2017) approaches, the relations between well-being and ecological, economic, and social sustainability can be characterized as follows (Figure 2): Human well-being is the intrinsically valuable outcome that the system aims to produce. Biophysical resources are the finite resources used to accomplish this well-being, setting the planetary boundaries for how this well-being can be accomplished sustainably. The economic, communal, and institutional systems in this analysis are the provisioning systems that transform biophysical resources and human labor into human well-being. The crucial question facing humanity in this era of Anthropocene is how to provide well-being for all humanity while remaining within planetary boundaries (Hickel, 2019; O’Neill et al., 2018; Randers et al., 2019; Raworth, 2017).

## Constraints on Generality—How Universal Is the Present Theory?

The ambitious aim (with emphasis on *aim*) of this paper is to build a universal model of human well-being, as establishing such psychological universals (Majid, 2023; Murdock, 1945; Norenzayan & Heine, 2005) would be crucially important for evaluating and comparing the well-being of people across the world and to establish what universal factors support human well-being (Berry, 2013; Mathews & Izquierdo, 2010). At the same time, as regards citations statement, while I did not extract information about all authors and samples given that the present article cites 282 past work, it is clear that, following the general trend in psychology, the overwhelming majority of empirical papers and previous theoretical models that the present theory builds on come from North America and Europe, and thus from contexts that are not representative of the global population (Apicella et al., 2020; Henrich et al., 2010a, 2010b). Given this, an attempt has been made to examine relevant cross-cultural evidence for all the major claims made in the article about, for example, human needs and well-being effects.^7^ There are many examples of proposed universals turning out to be artifacts of certain cultural contexts (see e.g., Henrich, 2020), but many universals have also stood the test of anthropological scrutiny (see Brown, 1991), making it paramount to evaluate how strong claims about universality can be made about various parts of the model.

On the most general level, we humans are biological, social, agentic, and experiencing beings no matter the particular situation or cultural context, and thus, our well-being is universally affected by concerns and needs related to having, loving, doing, and being. However, when we move to more concrete indicators and need satisfiers, and when we examine how much various people value these dimensions, we start to find much cultural variation.

One area where cultural variation is prone to exist is on who is the “self” making evaluations about its well-being and need satisfaction. While human experiences are subjective, human selves and identities are always more or less relational (Gergen, 2009; Helne, 2021), as humans derive their sense of self partly by identifying with their social groups (Tajfel & Turner, 1986). This has led to important work on collective psychological need satisfaction (Greenaway et al., 2016; Kachanoff et al., 2020), examining how people’s personal need satisfaction and well-being are impacted by the degree of collective autonomy (Kachanoff et al., 2019, 2021), collective competence and efficacy (Oyserman et al., 2006; see also Fryberg et al., 2008), and collective relatedness (Branscombe et al., 1999). Given that people in modern Western societies have historically the most individualistic, independent, and narrow self-concept (Henrich et al., 2010b; Markus & Kitayama, 1991), we can expect the situation and need satisfaction of the collective to have a stronger impact on well-being in more collectivistic societies. For example, African psychology emphasizes *botho* and *ubuntu* as expressions of human interconnectedness and how personhood grows from and exists within social relations (Adjei, 2019; Sodi et al., 2021). Furthermore, identity can expand beyond the social realm, for example, many indigenous groups strongly identify with the environment and land they live in (Mark & Lyons, 2010; O’keefe et al., 2022), making threats to such factors also salient for their personal well-being (Ullrich, 2019). People in different cultures can thus have more restricted or expanded identities and selves, influencing whom and what they take into account when evaluating their need satisfaction and well-being.

However, despite various identified cultural differences (see, e.g., Kitayama et al., 2022; Vignoles et al., 2016), a global science of well-being seems possible, given our shared human nature (Mathews & Izquierdo, 2010; Veenhoven, 2012; Zwolinski, 2019). While some cultural differences in using the scales have been identified (e.g., Angelini et al., 2014; Brulé & Veenhoven, 2016), most cross-cultural variance in well-being ratings are seen to be resulting from real differences in life conditions, with different cross-cultural surveys painting a relatively consistent picture of factors influencing the well-being of nations (Veenhoven, 2008; Zwolinski, 2019). Basic well-being–related indicators have been successfully translated and utilized across the world in more than 150 nations by organizations such as Gallup and World Values Survey and by various independent research groups, demonstrating that most well-being indicators make sense and can be used with people from different cultures. In addition, the cross-cultural comparisons of how various needs contribute to well-being have mostly supported their universality (Aknin et al., 2013; Chen, Vansteenkiste, et al., 2015; Church et al., 2013; Martela et al., 2023; Tay & Diener, 2011), while Fischer and Schwartz (2011, p. 1127) concluded, based on a cross-cultural study with over 60 countries, that “values associated with autonomy, relatedness, and competence show a universal pattern of high importance and high consensus.”

So the target population (Simons et al., 2017) of the present theory of well-being is the whole human population, even though it is acknowledged that any attempt to build such a “universalist vision for psychology” (Berry, 2013, p. 395) will have its blind spots and unidentified constraints emerging from the particular background(s) of the theorists (Fernando & Moodley, 2018). Finding the universal factors of well-being that unite us all is an important task, and we should not shy away from trying (Berry, 2013; Jensen, 2012; Thin, 2010). Accordingly, the present work ought to be seen as one particular voice inviting others into a dialogue where many more voices—and a more diversified set of voices—are needed to together take steps toward identifying the shared common ground of human well-being.

## Discussion

To measure and advance well-being, we need well-grounded theories of well-being. A theory of well-being is ultimately a theory about human nature and human needs—what is it like to have a good life for a human being? As Galtung argued, “basic needs approaches are indispensable in any theory of development that sees development as development of human beings” (Galtung, 1980, p. 104). Thus, to understand human well-being, we need to understand what existence is like for us humans.

Accordingly, building on the work of Erik Allardt (1976, 1993), later extensions of his theory (Helne & Hirvilammi, 2015; Hirvilammi & Helne, 2014), and more recent research on subjective well-being (Diener et al., 1999; Diener, Oishi, & Tay, 2018) and psychological needs within the self-determination theory (Ryan & Deci, 2000, 2017), I argued that human existence, and thus human well-being, is multi-dimensional, identifying four modes of existence for humans: *Having* recognizes us as material-biological beings with physical needs, *loving* recognizes us as social beings with social needs, *doing* recognizes us as active beings with agentic needs, and *being* recognizes us as beings who experience their existence, associated with affective and evaluative well-being.

As regards psychological research on the nature of well-being (Diener, Lucas, & Oishi, 2018; Huppert & So, 2013; Keyes, 2007; Martela & Sheldon, 2019; Ryff & Keyes, 1995), the present work contributes to the debate about what it is and how it should be measured by providing an integrative theory of well-being that anchors the proposed dimensions in human nature and human modes of existence and provides a novel way of categorizing various previously proposed indicators. The present theory contributes especially to the ongoing debate on how life satisfaction and affects (what Diener calls subjective well-being, Diener, 1984) relate to more eudaimonic and functional dimensions of well-being (Huppert & So, 2013; Keyes, 2007; Ryff, 1989). Here it is argued that psychological needs are at the core of more eudaimonic and functional dimensions of well-being and ought to be seen as important indicators of well-being as such—but also key factors contributing to affective and evaluative well-being (Martela & Ryan, 2023; Martela & Sheldon, 2019). Thus, the present work goes beyond the dichotomy between “functional/eudaimonic” well-being and “subjective/hedonic” well-being by arguing for the importance of both, while offering a clarification of how they relate to each other: The former are key factors anchored in human nature, the satisfaction of which significantly affect the latter. Causally, it is proposed that the satisfaction of the needs related to having, loving, and doing will lead to a higher sense of perceived well-being. The present work aims also to resolve the ambiguity and lack of unification around functional/eudaimonic dimensions of well-being (Heintzelman, 2018; Martela & Sheldon, 2019; Stone & Krueger, 2018) by proposing that they should be anchored in human physical, social, and agentic needs.

Besides offering the theory of four modes of existence as the core of well-being, the present work also integrates the rich psychological research tradition on the subjective dimensions of well-being into the broader discussions in philosophy, economics, and development studies on how well-being as such ought to be conceptualized and measured. Here, it aims to address the more philosophical critiques against psychological research as having operationalizations of well-being without a theory of well-being (Alexandrova, 2017; Fabian, 2022b) by strengthening the theoretical grounding of subjective dimensions of well-being. The present inquiry thus aims to integrate objective and subjective well-being traditions and indicators into one theory, with human needs and modes of existence as the common denominator.

As compared to the original contribution by Allardt, who saw needs as “socially defined” (Allardt, 1973b, p. 64), and the later extensions (Helne & Hirvilammi, 2015; Hirvilammi & Helne, 2014), the present work goes beyond them by integrating the proposed dimensions more deeply into human nature through more extensive discussion of their anchoring in modes of existence and through integrating them with more contemporary research on human needs. In defining needs, the present theory draws especially from the self-determination theory and the explicit criteria for human needs developed therein (Martela & Ryan, 2023; Ryan & Deci, 2017). Having explicit criteria for what counts as a human need makes it possible to identify and evaluate potential needs empirically. The present work builds on needs-based accounts of well-being (Doyal & Gough, 1991; Galtung, 1980) but comes to offer a new proposal about key needs and, through the modes of existence, a new way of making distinctions within them and anchoring them to human nature. Given that Allardt was inspired by Maslow, these same points apply also as to the differences between the present theory and Maslow’s (1943, 1954) theory of needs: The present theory offers a different list of needs and anchors the needs in modes of existence, while not proposing a strict hierarchy of needs. Besides updating Allardt’s theory by integrating it with more recent developments in the science of well-being, the present theory also offers, compared to Allardt (and Maslow), a completely new conceptualization of *being* as tapping into humans as experiencing creatures. This helps to anchor affective and evaluative well-being into the theory and clarifies how affective and evaluative dimensions of well-being relate to the more needs-based accounts of well-being.

As regards previous need theories, human social needs are recognized by virtually all need theories, whether it is called relatedness (Alderfer, 1969; Ryan & Deci, 2017), the love needs (Maslow, 1943), the need to belong (Baumeister & Leary, 1995), the need for affiliation (McClelland, 1985), or the need for communion (Bakan, 1966). Here, the present work identifies three potentially separate social needs for inclusion, relatedness, and prosociality. As regards the agentic needs for autonomy and competence, they are recognized within the self-determination theory (Ryan & Deci, 2017) but not in other need theories (although competence is hinted in McClelland’s, 1985, need for achievement and Doyal & Gough, 1991, propose a need for autonomy but define it quite differently). Interestingly, save for Maslow (1943), physical needs and safety are rarely examined in such need theories (although briefly discussed by Ryan & Deci, 2017). Compared to these previous theories, through integrating Allardt and the self-determination theory, the present theory thus provides a novel conceptualization of basic human needs.

### Future Directions

Any theory of human modes of existence and needs must remain open to be revised in the future, something most need theorists have always emphasized (Allardt, 1976; Galtung, 1980; Martela & Ryan, 2020; Ryan & Deci, 2017). An inquiry into the basic elements of well-being, as Doyal and Gough (1991, p. 141) argue “must bring to bear *both* the codified knowledge of experts and the experiential knowledge of those whose basic needs and daily life world are under consideration.” This entails a certain back-and-forth movement between universal syntheses of human well-being and needs and more localized attempts to identify what well-being means for particular groups in particular historical situations (Sollis et al., 2022). In this regard, there have been many interesting local projects where participants have had a voice to identify good life and well-being in that group (see Gough, 2020; Sollis et al., 2022). Combining such projects with more research-based inquiries into what humans strive for (e.g., Benjamin et al., 2014) and what are the key sources of suffering and wellness in humans—in particular, cross-cultural research examining how broadly the proposed dimensions of well-being generalize (e.g., Martela et al., 2023)—will (and should) challenge and improve the current theory, ensuring a more empirically-grounded and universal theory of well-being in the future.

The proposals made here should thus stimulate empirical research aiming to refine and challenge various parts of the theory. One open question is the role of human attitudes and ways of approaching life in a theory of well-being. I have focused on *being* as an experiencer of well-being. However, humans are also more active interpreters of the world, with our attitudes, beliefs, and norms significantly shaping how we perceive the world, how we approach it, and how we behave within it—and all these have consequences for our well-being. Accordingly, optimism, mindfulness, self-esteem, and self-acceptance have from time to time been proposed as dimensions of well-being (e.g., Huppert & So, 2013; Marsh et al., 2020; Ryff, 1989), while there has also been interesting research on, for example, the role of self-compassion (Neff, 2011) and quiet ego (Bauer & Weatherbie, 2023) in well-being. A theory of what such basic well-being-conducive attitudes would be and how they would be integrated into a broader theory of well-being is still lacking. Here, the focus has been on the dimensions of *being* that are most directly about well-being and most universal, hence a focus on affective and evaluative well-being. However, how human attitudes and human active inference of the world influence and are related to well-being is a task for future research. Similarly, it is a task for future research to investigate whether psychological richness (Oishi & Westgate, 2022) should be integrated as an additional element of *being.*

Note the absence of spirituality and religion in the present model of well-being. Majority of the world’s population affiliate with some religious tradition (Pew Research Center, 2012), and for many of them, religiosity and spirituality are key dimensions for well-being (Tarakeshwar et al., 2003), with research suggesting that some aspects of them have small, positive associations with well-being (Garssen et al., 2021; Poloma & Pendleton, 1990; Witter et al., 1985). However, cross-cultural research has produced more mixed results, demonstrating how cultural context seems to significantly influence the relationship between religiosity and well-being (Lavrič & Flere, 2008; Martela et al., 2023). Furthermore, there is considerable variety in how religiosity and spirituality are expressed in different cultures (Ruiter & Van Tubergen, 2009; Saroglou et al., 2020), making it hard to capture what the more universal aspects of the phenomenon are. Also, the positive association between religiosity and well-being is seen to be much dependent on cultural norms (Hoogeveen et al., 2022). The expressions of religiosity and spirituality thus seem to be so deeply embedded in specific cultural contexts that more research is needed to capture what is universal about religion and spirituality (see Norenzayan et al., 2016; Saroglou, 2011) and whether there are aspects of religiosity contributing to well-being across cultural contexts. Accordingly, the absence of religion in the current model is not an expression of it not being important for well-being but a call for future research to better capture its key aspects and potential relations with well-being.

Similarly, sexuality is currently not discussed, even though sexual activity is a universal human behavior (Brown, 1991), necessary for the survival of our species, and often a strongly motivating and satisfying act, with satisfying sexual lives having positive well-being consequences (Diamond & Huebner, 2012; Muise et al., 2016). However, there is much cultural variation in how sexuality is expressed (Brown, 1991), many people live long periods seemingly happy without sexual activity, and there are debates about what forms of sexual activity are beneficial (Diamond & Huebner, 2012), making the role of sexuality in human well-being a complex topic that would require more work.

It is also worth noting that in taking human well-being as the target of inquiry, the present work can be seen as anthropocentric. Recently, there have been discussions about less human-centric notions of well-being that aim to better take into account human interrelatedness with the broader and natural world, leading to notions such as planetary well-being (Kortetmäki et al., 2021). Integrating the present theory of human well-being with more holistic and relational views on well-being (e.g., Helne, 2021; S. C. White, 2017)—by, for example, expanding *loving* to include belonging to and caring about the natural world beyond humans—remains another area for further development.

Furthermore, the flagship indicators of subjective well-being have tended to focus on *averages* among various groups. While averages and medians convey important information, for many societal purposes, it is more relevant to identify minimum thresholds and monitor how many people are above it. As Allardt (1993, p. 90) notes: “More important than dispersion measures, however, is the notion of a floor, a bottom level, below which no individual should be located.” Thus, future research should identify whether averages, medians, or minimum thresholds are most informative for various indicators. Finally, while the present work has offered a proposal about the constructs to be measured within each mode of being, the selection of exact indicators for each construct is a task for the future. The author hopes that the present theoretical synthesis does not close down inquiry but rather presents a platform upon which many future inquiries can build.

### Practical Implications

For a theory of well-being, the practical implications emerge from whether and how it is used. A fundamental purpose of public policy is “to protect and promote the well-being of citizens” (Boarini et al., 2014, p. 10), and countries around the world have started increasingly ambitious initiatives to measure well-being, with the more progressive governments aiming to integrate the results more into their political processes (Exton & Shinwell, 2018). The more we know about the factors that generate well-being on an individual level, on a regional level, and on a national level, the more this enables evidence-based policy that serves citizen well-being. For example, given increased skepticism about the possibility of “green growth” where economic growth is decoupled from a negative environmental impact (Hickel & Kallis, 2020), it is good to realize that economic growth seems to deliver diminishing returns of investment—beyond a certain point, additional economic growth has either a marginal (e.g., Stevenson & Wolfers, 2013) or a non-existent (e.g., Jebb et al., 2018) relation with well-being. However, many non-material factors such as the quality of democracy (Helliwell & Huang, 2008; Ott, 2011), gender equality (Audette et al., 2019), and a sense of trust between people (Delhey & Dragolov, 2016; Helliwell, Huang, & Wang, 2018) have been shown to be crucially important for national levels of well-being, thus providing more environmentally sustainable and more robust ways of increasing the well-being of citizens across the world. More comprehensive assessment of well-being enabled by stronger theory of well-being provides a yardstick and a target for politics and allows policy-makers to identify more effective and sustainable ways to improve the well-being of the citizens in the future.

Currently, however, subjective well-being measurement in policy use is “almost entirely centred on life satisfaction” (Mahoney, 2023, p. 21). While measurement of positive and negative affect has started to become more prevalent, OECD (2013), Eurostat (2017), and the US National Research Council (2013) have all recommended measuring also more functional/eudaimonic dimensions of well-being. Thus far, this has been hard to implement given the ambiguity around what the functional dimension of well-being entails (Heintzelman, 2018; Martela & Sheldon, 2019; Stone & Krueger, 2018). Here, the present framework with its list of physical, social, and agentic needs can help in choosing the most relevant and universal functional dimensions of well-being to be measured alongside life satisfaction to build a more comprehensive subjective well-being assessment. Such harmonization is crucially needed, as it would allow comparability and a more cumulative and global science of well-being (Mahoney, 2023).

## Conclusion

The most pressing challenge for humanity, right now, is to offer adequate well-being for people in a way that ensures that the planet remains habitable also in the future (Fanning et al., 2022; O’Neill et al., 2018). Well-being provides the ultimate societal goal, and the ecosystem, the uncompromisable limits within which this goal has to be reached. Economic growth, in this scheme, has no intrinsic value, as economic indicators only capture features of the “provisioning system” that aims to transform natural resources and human labor into well-being (O’Neill et al., 2018). Unfortunately, fixation on economic growth and GDP as key indicators of national progress has confused ends and means and is increasingly recognized as one key obstacle in the path toward more sustainable production of well-being (Costanza et al., 2014; Hoekstra, 2019; Stiglitz et al., 2009, 2018). To bypass the domination of economic growth as a *de facto* target in political decision-making, we need more established, theory-grounded, and methodologically sound indicators of well-being that could take the role of key targets of political decision-making and measures of national progress and development.

The present article aims to contribute to this task by clarifying what well-being for humans means, aiming to offer a comprehensive account of well-being grounded in human nature, identifying four modes of existence—*having, loving, doing*, and *being*—and proposing key indicators for each of them. Better theories of well-being make possible better measurement of well-being, which makes possible more effective and evidence-based advancement of human well-being. The present article ultimately aims to offer its small contribution to this advancement of the well-being of humans on planet Earth.

## Notes

1. Allardt identified *having, loving*, and *being* as the three need categories. In aligning his work with more recent research on well-being, I come to propose renaming what he called *being* as *doing*, to focus it more clearly on human active nature, while I propose *being* as a new mode of existence focusing on humans as experiencing their own lives, thus capturing affective and evaluative well-being.

2. It is worth noting that there are important cross-cultural differences in how people acquire and come to value their goals and aspirations. Cultures with more independent self embrace a more disjoint model of agency emphasizing self-selection and individual desires and goals, while cultures with more interdependent self embrace a more conjoint model of agency emphasizing responding to obligations and role expectations (Markus & Kitayama, 2003). So, while “purposeful agency appears to be universal” (Markus & Kitayama, 2003, p. 4), the kind of values and self the persons aim to fulfill through their agency can vary.

3. For Allardt, *being* was a need category “with alienation at one extreme, and self-actualization at the other” (Allardt, 1973a, p. 13), but he also briefly mentioned “Doing” as one part of it (Allardt, 1993). Taking inspiration from Hirvilammi and Helne (2014), who made a distinction between *being* and *doing*, I have here taken the liberty of renaming what Allardt called *being* into *doing*, as I see that the essence of it is to capture the agentic dimension of human existence, the fact that “humans are inherently active beings” (Hirvilammi & Helne, 2014, p. 2166), and thus *doing* is a more accurate label for it than *being*.

4. Kahneman calls these the experiencing self and the remembering (or evaluating) self, when contrasting actual experiences to our memories about them (Kahneman & Riis, 2005). But I see that conscious reflection is one part of the human way of experiencing, and thus, we need a more specific label for the first. Hence, “engaged self” denotes the emotionally charged attunement to the current situation. Furthermore, conscious and deliberate information processing involves more than memory, making “reflective self” a better label for it.

5. Kahneman and Riis call these *experienced well-being* and *evaluative well-being*, and these labels have been adapted by the National Research Council among others. However, “experienced well-being” is a misleading label because even our evaluations are part of our overall experience (Dewey, 1939), and thus, I prefer “affective well-being” as the more accurate label for the “Type I well-being.”

6. While the exact number of cross-culturally universal basic emotions is still discussed, the distinction between positive and negative emotions is considered universal (Ekman, 1992; Elfenbein & Ambady, 2002). There is also cross-cultural variation in norms for experiencing emotions, with some types of emotions more valued (e.g., pride in individualistic cultures, guilt in collectivistic cultures), and some others more disvalued in certain cultures (e.g., negative affect in certain African cultures; Eid & Diener, 2001; Kim-Prieto & Eid, 2004). However, such differences in how specific emotions are valued do not challenge the universality of people preferring to experience positive rather than negative emotions in life (Heine, 2020; Zwolinski, 2019).

7. While such cross-cultural work has mainly supported the conclusions made, even that work tends to focus on the wealthy, urban, college-educated populations of their target countries, leading to blind spots about how well certain conclusions would generalize to, for example, various indigenous populations.
